# Conventional scan body vs. scan bodies with auxiliary geometric devices: an *in vitro* study for edentulous full-arch implant impressions

**DOI:** 10.3389/froh.2025.1574149

**Published:** 2025-06-02

**Authors:** Francesco Gianfreda, Carlo Raffone, Mirko Martelli, Alberto Pitino, Vito Carlo Alberto Caponio, Patrizio Bollero

**Affiliations:** ^1^ Department of System Medicine, University of Rome “Tor Vergata”, Rome, Italy; ^2^ Independent Researcher, Rome, Italy; ^3^ Department of Clinical Science and Translational Medicine, University of Rome “Tor Vergata”, Rome, Italy; ^4^ Independent Researcher, Turin, Italy; ^5^ Department of Life Sciences, Health and Health Professions, Link Campus University, Rome, Italy

**Keywords:** full-arch, digital impression, scan body, digital implantology, digital dentistry

## Abstract

**Aim:**

This study aimed to assess the effectiveness of an auxiliary geometric device (AGD) in enhancing the trueness of full-arch implant impressions. The primary metrics of interest were total surface deviation (TotRMS), centroid deviation (cRMS), and angular deviation. All these values are crucial for achieving a precise fit of implant-supported prostheses.

**Methods:**

A gypsum-based edentulous maxillary model with four multi-unit abutment replicas was prepared, replicating clinical scenarios. Control and experimental scan bodies were scanned using an intraoral scanner (Dexis 3800), and the resulting data were compared to a digital master model created with a structured light scanner (ATOS compact Scan 5M). The AGD was used to reduce positional errors during the scan process. Data were processed using Exocad and GOM Inspect Professional software, aligning scan body library files with mesh data using a best-fit algorithm. Angular, platform, and total deviations were calculated to assess positional trueness. A sample size of 20 scans per group was determined *a priori*, and statistical comparisons were made using Mann–Whitney *U* tests.

**Results:**

The inclusion of the AGD significantly reduced centroid root mean square (cRMS) values in all measured comparisons (*p* < 0.001), demonstrating enhanced trueness. The total body root mean square deviation **(**TotRMS) values also showed a significant reduction (*p* = 0.002). While overall angular deviation differences were not statistically significant (meanAGD = 0.38; meanNO = 0.39; where “NO” refers to the group without AGD), site-specific analysis revealed significant improvements at points 2.4 (*p* = 0.017) and 1.4 (*p* < 0.001). The Euclidean distance in platform deviation consistently indicated better alignment in the AGD group.

**Conclusion:**

The AGD, tested under *in vitro* conditions, significantly improved the trueness of full-arch implant impressions, particularly reducing c- and TotRMS values. These findings highlight the AGD's potential to enhance digital workflows in implant dentistry by mitigating positional discrepancies and ensuring greater trueness and precision. Future research should explore these findings in a clinical scenario.

## Background

Full-arch implant-supported rehabilitations provide an effective solution for edentulous patients, offering improved function, esthetics, and quality of life (1). However, the clinical success of these treatments depends heavily on the precision of the impression process (2). An accurate transfer of implant positions to the virtual or physical model is essential for ensuring a passive fit of the prosthetic framework (3). Misfits caused by inaccuracies in impressions can result in mechanical complications, such as screw loosening, fractures, and stress on the peri-implant bone, ultimately compromising long-term outcomes (4, 5).

The advent of digital workflows has transformed implant dentistry, with intraoral scanners (IOSs) and computer-aided design and computer-aided manufacturing (CAD/CAM) technologies becoming integral components of modern practice. By eliminating traditional impression materials, digital techniques reduce patient discomfort and minimize errors associated with material shrinkage or expansion (6, 7). Central to the digital impression process are scan bodies, which are used to transfer the three-dimensional position of implants to the virtual model. While these devices are reliable in single-unit or short-span cases, their performance in full-arch edentulous scenarios has significant limitations (8).

Full-arch scanning in edentulous patients presents unique challenges due to several factors. The absence of natural teeth eliminates anatomical landmarks, which are critical for maintaining spatial orientation and minimizing stitching errors during scanning (9). Additionally, edentulous ridges often exhibit uneven, soft tissue contours, leading to inconsistent scan data capture (10). The lengthy span between implants in full-arch cases exacerbates stitching errors, as slight inaccuracies in aligning overlapping scans accumulate over the arch (11). This results in cross-arch distortions that can compromise the trueness and precision of the final model (2).

Moreover, conventional scan bodies lack mechanisms to stabilize their orientation during the scanning process, further contributing to angular deviations and positional inaccuracies (12, 13). The variability in scan body geometry, scan body-library mismatches, and the influence of operator-dependent factors, such as scanning technique and sequence, further amplify these limitations (14–16). These challenges collectively make achieving a clinically acceptable impression more complex in full-arch edentulous cases compared to partially dentate situations.

To address these shortcomings, auxiliary geometric devices (AGDs) have been introduced as an adjunct to traditional scan bodies (17). AGDs aim to enhance scan body stability and improve alignment during scanning, potentially reducing stitching errors and improving overall accuracy (18, 19). However, comprehensive evidence comparing the performance of conventional scan bodies with and without AGDs remains limited.

This study aimed to evaluate the impact of auxiliary geometric devices on the trueness of edentulous full-arch implant impressions. Using an *in vitro* model, conventional scan bodies were compared with those incorporating AGDs, assessing trueness and precision metrics, including centroid root mean square (cRMS) and angular deviations.

## Methods

**Figure 1:**
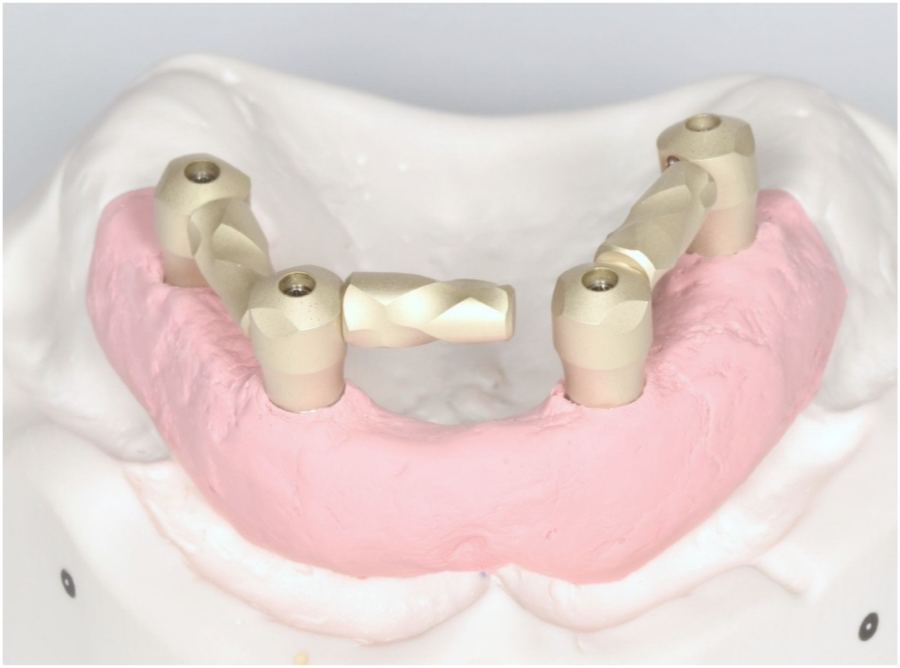
Test scan body with the AGD.

**Figure 2:**
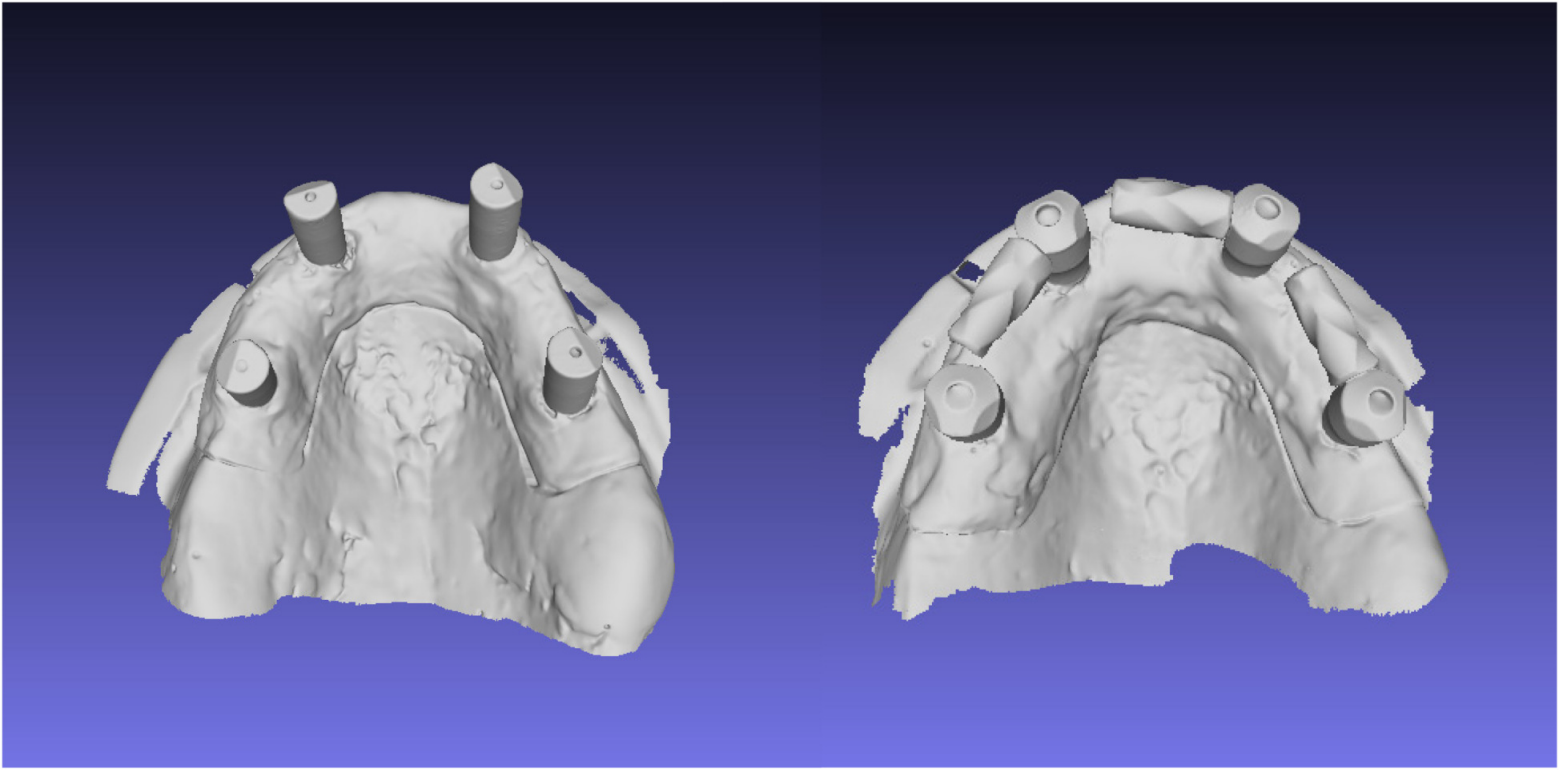
Control scan body and experimental (test) scan body digitized with an IOS.

**Figure 3:**
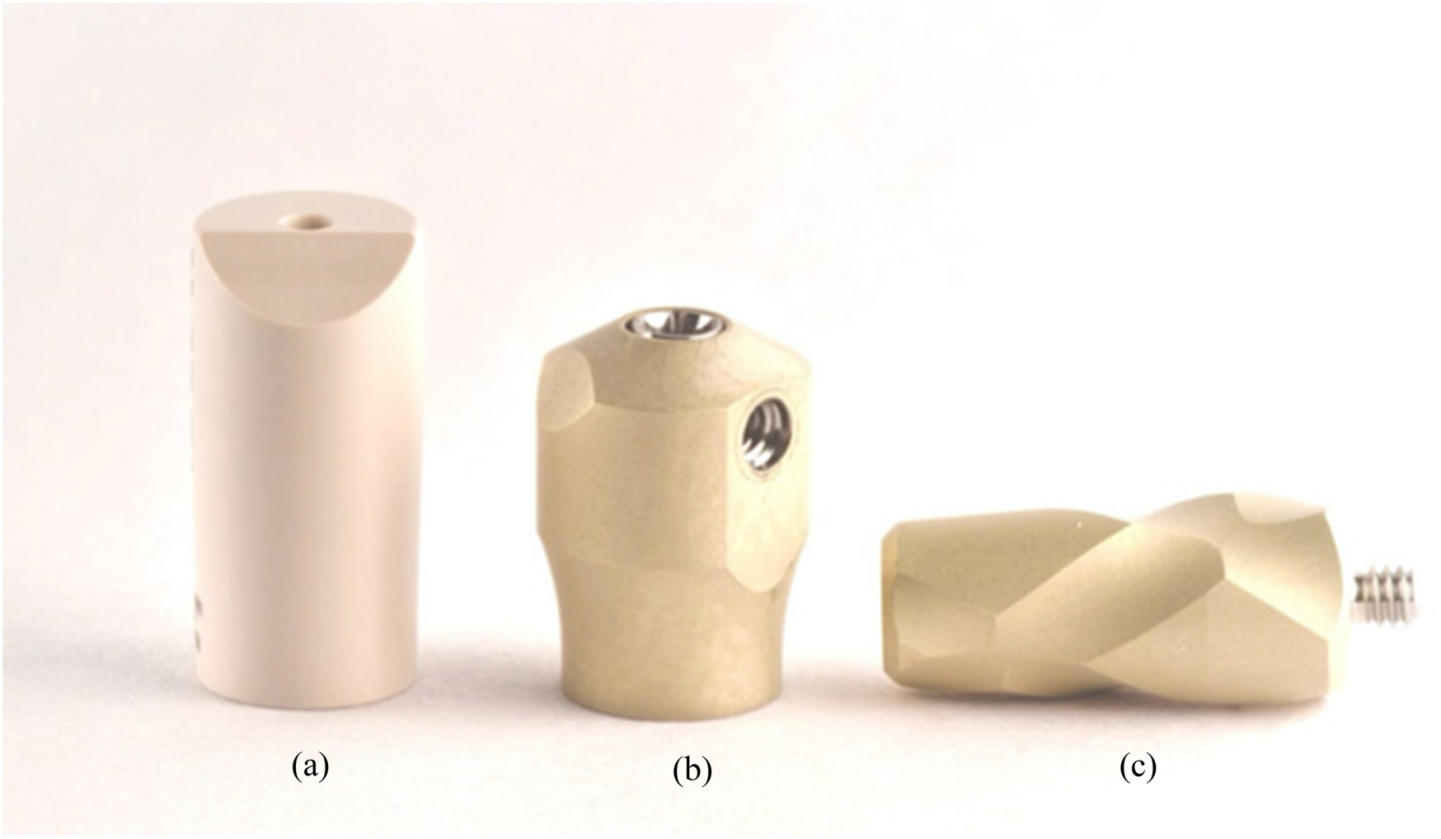
Close-up of the conventional scan body **(a)** and the test scan body **(b)** with the AGD **(c)**.

This experimental *in vitro* study, designed to assess the accuracy of digital full-arch implant impressions in an edentulous maxillary model, was conducted in December 2024 at the University of Rome “Tor Vergata,” Italy. The acquisition of the master model using an industrial optical structured light scanner, was performed at Measure3D, Rome, Italy. An edentulous maxillary model was realized using gypsum with four multi-unit abutment (MUA) replicas (Nobel Biocare AG, Kloten, Switzerland) at the lateral incisor and second premolar sites. The inter-implant distance was 16.5 mm between the implants in tooth sites 1.4 and 1.2, 16.7 mm between sites 1.2 and 2.2, and 16.6 mm between sites 2.2 and 2.4. Control scan bodies (Nobel Biocare AG, Kloten, Switzerland) 9.5 mm in height and 5 mm in diameter and test scan bodies 7 mm in height and 6 mm in diameter with AGDs 8, 10 and 12 mm in length (Nobel Biocare AG, Kloten, Switzerland) were screwed directly on the MUA replica (Figures 1, 2). The main differences between the two scan bodies concern the material and shape. Specifically, the test scan bodies differ by being made of metallic material instead of polyetheretherketone (PEEK), and especially due to the presence of a screwable extension that creates an L-shaped scan body (Figure 3). This component was designed to reduce the scanning area exclusively related to soft tissues, thereby facilitating the stitching of images during acquisition.

**Figure 4:**
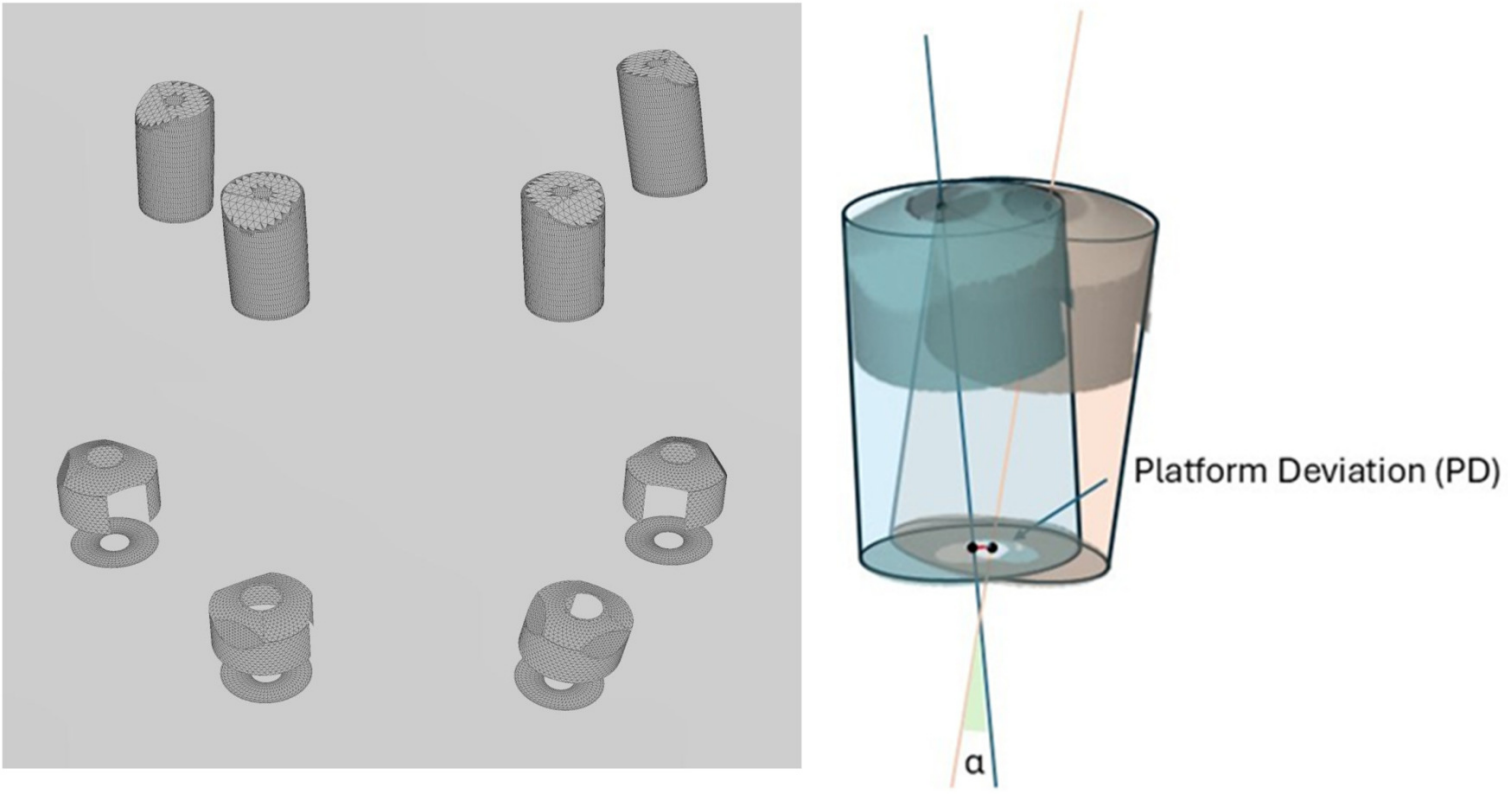
Meshes of the scan bodies exported from the library and used for superimposition and deviation analysis. Illustration of the platform deviation (PD) and angular deviation (*α*) measurements.

**Figure 5:**
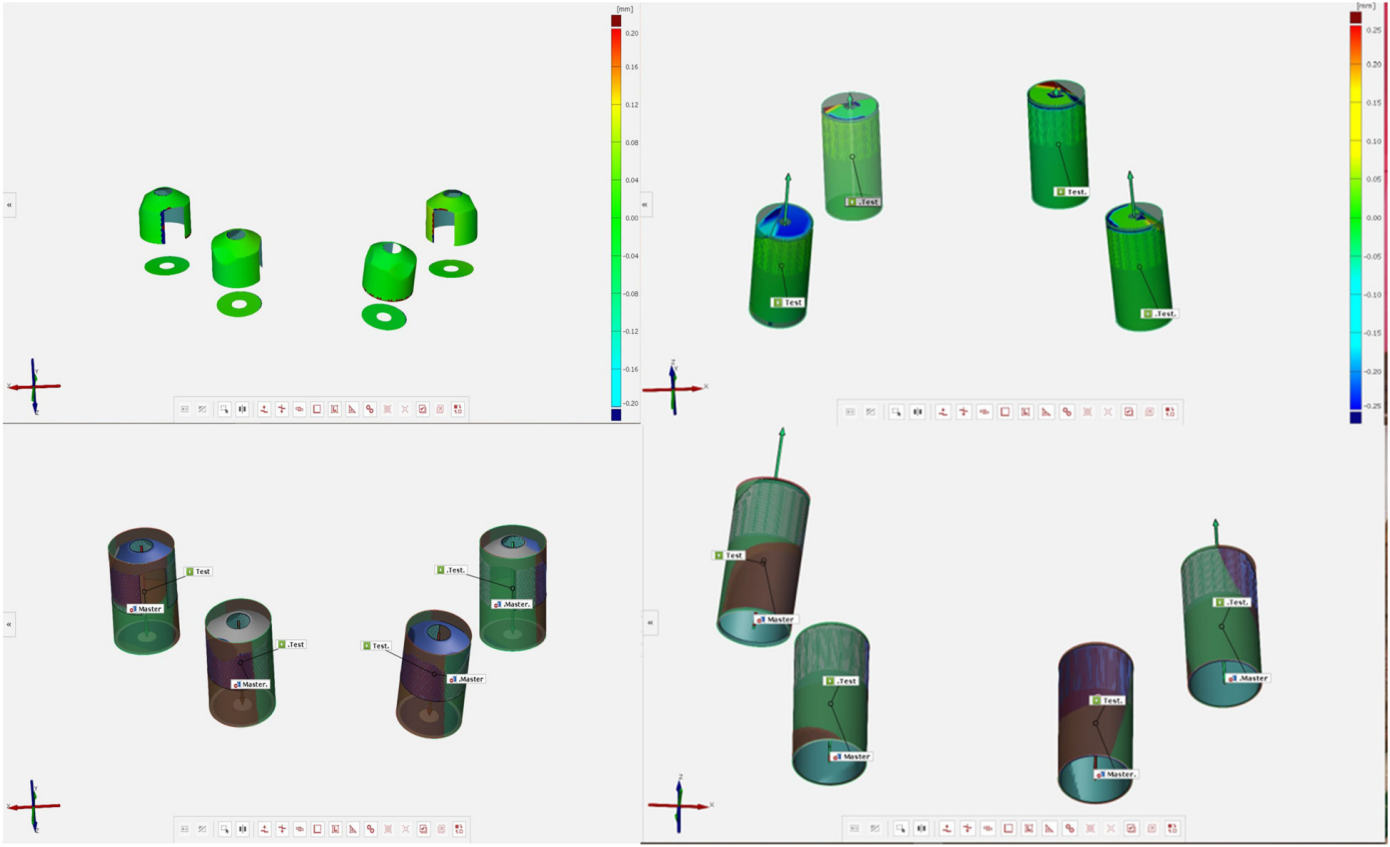
Surface deviation and platform deviation of the experimental and test scan bodies.

**Figure 6:**
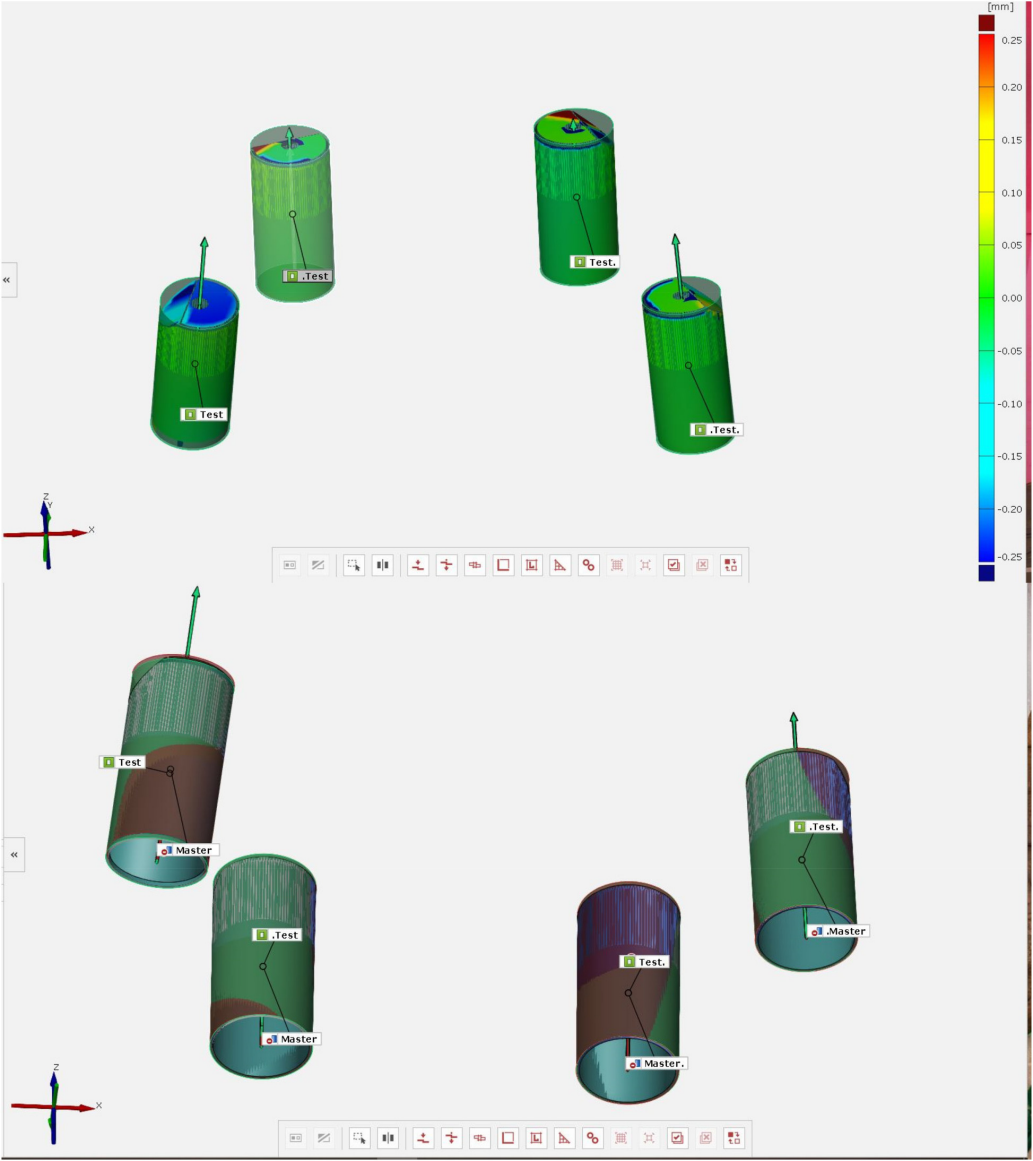
Surface deviation and platform deviation of the test scan bodies.

### Digital scanning

Intraoral scanner (Dexis 3800) (Dexis, Quakertown, Pennsylvania, United States) was used following the manufacturer's instructions for the scan strategy, progressing from the occlusal surface to the palatal and buccal surfaces. Scans were taken with the control scan bodies and the experimental ones. A digital master model was created using an industrial optical structured light scanner (ATOS compact Scan 5M, GOM GmbH, Braunschweig, Germany), with a trueness of 3 µm and a precision of 1.7 µm (20). Each experimental scan was imported as a standard tessellation language (STL) file into dedicated software (Exocad, GmbH, Darmstadt, Germany) to align the scan body library with the mesh data using a best fit algorithm.

### Digital analysis

The aligned scan bodies from the library were then exported as STL files to inspection software (GOM Inspect Professional, GOM GmbH, Braunschweig, Germany) for the final surface comparison (Figure 3). The master scan was selected as the CAD geometry and individually aligned with each experimental scan using the pre-alignment tool. The “CAD comparison” tool was used to perform a “surface comparison on actual elements.” A fitting cylinder was aligned on the cylindrical lower part of each scan body and the center of the base (centroid) and the axis were determined. Angular deviation was calculated as the angle between the axes of the two cylinders. Platform deviation (PD) was calculated as the Euclidean distance from the corresponding centroid. The lower part of each scan body and the center of the base (centroid) and the axis were determined. Angular deviation was calculated as the angle between the axes of the two cylinders.

### Sample size calculation and statistical analysis

The *power two means* command in STATA 14.0 was used to calculate the sample size *a priori*. The root mean square (RMS) trueness difference between using an AGD and not using an AGD on the entire scan body was taken into consideration when calculating the *a priori* sample size. The study of Wu et al. provided RMS trueness means in the scanned models with an AGD (67.44 µm) and standard deviation (SD) of 9.63 µ compared to the no AGD group (mean of 82.29 µ and SD of 17.31 µ) (21). A total of 20 scans per group were performed, exceeding the minimum requirement of 16 scans per group identified by the *a priori* power analysis to achieve 80% power with an alpha of 0.05.

The RMS trueness values for the whole body and the centroid and the angular deviation were recorded at four different points. Their normality distribution was explored on each point and as an average (Shapiro–Wilk *p*-value < 0.001). Due to the non-normal distribution, differences in these linear variables between the AGD and no AGD groups were investigated using the Mann–Whitney *U* test.

## Results

Three primary outcomes were assessed to evaluate the accuracy of full-arch implant impressions: cRMS, total body root mean square deviation (TotRMS), and angular deviation. The use of the AGD resulted in a statistically significant reduction in cRMS values across all measured implant sites (2.4, 2.2, 1.2, and 1.4), with *p*-values < 0.001 for each comparison (Table 1). The average cRMS in the AGD group was 48.13 µm, compared to 244.13 µm in the control group, indicating a substantial improvement in positional accuracy (Table 1). In terms of TotRMS, the AGD group also showed a significant reduction in average values (33.38 µm with AGD vs. 67.63 µm without AGD; *p* = 0.002) (Table 1). When analyzed by individual site, the difference was statistically significant at position 1.2 (*p* < 0.001), while the remaining sites showed consistent, although not statistically significant, reductions. Regarding angular deviation, the overall average values were similar between the two groups (AGD = 0.38°, NO AGD = 0.39°; *p* = 0.327) (Table 1), and both remained well within the clinically acceptable threshold of 1°. However, site-specific analysis revealed significant differences; at site 1.4, the AGD significantly reduced angular deviation (*p* < 0.001), whereas at site 2.4, the deviation was unexpectedly higher in the AGD group (*p* = 0.017) (Table 1, Figures 4–6). These results collectively suggest that the AGD enhances the trueness and consistency of full-arch implant impressions, particularly by minimizing centroid and total body deviations. While the impact on angular deviation was not uniform, localized improvements further support the AGD's role in stabilizing scan bodies and mitigating positional errors.

**Table 1:** Measurements (µm) with (test group) and without (control group) AGD.

Auxillary device (µm)							
Variables	Control group		Test group with the AGD		Mean difference	Std. error difference	*p*-value
	Mean	Standard deviation	Mean	Standard deviation			
A_dev2.4	250	126	402	229	−153	58	0.017
A_dev2.2	336	134	578	752	−243	171	0.883
A_dev1.2	236	138	253	161	−17	47	0.779
A_dev1.4	740	379	293	145	447	91	<0.001
CRMS2.4	268.5	175.3	43.0	18.7	225.50	39.41	<0.001
CRMS2.2	258.5	229.4	78.5	107.5	180.00	56.65	<0.001
CRMS1.2	261.0	174.5	31.5	10.4	229.50	39.08	<0.001
CRMS1.4	188.5	163.1	39.5	19.0	149.00	36.71	<0.001
BRMS2.4	70.5	51.5	44.5	34.4	26.00	13.84	0.091
BRMS2.2	66.0	58.2	29.5	11.9	36.50	13.28	0.081
BRMS1.2	80.5	57.0	21.0	14.8	59.50	13.17	<0.001
BRMS1.4	53.5	44.2	38.5	19.5	15.00	10.80	0.512
avgBRMS	67.63	40.53	33.38	9.36	34.25	9.30	0.002
avgCRMS	244.13	162.66	48.13	28.60	196.00	36.93	<0.001
avgDev	0.39	0.14	0.38	0.22	9	59	0.327

A_dev, angular deviation; CRMS, centroid root mean square deviation; BRMS, body root mean square deviation; avgCRMS, average CRMS across sites; avgBRMS, average BRMS across sites; avgDev, average angular deviation.

## Discussion

This study demonstrates the significant impact of an AGD (Figure 1) on improving the trueness of full-arch implant impressions in edentulous patients.

The findings of this study further align with the tolerance thresholds documented in the literature, which establish a maximum acceptable linear deviation of 150 µm (22) and an angular deviation of 1 degree for clinical applicability in implant-supported rehabilitations (23). Notably, the experimental group utilizing the AGD consistently achieved cRMS and TotRMS values significantly below the linear deviation threshold, with mean values of 48.13 µm and 33.38 µm, respectively. These results underscore the AGD's effectiveness in enhancing positional accuracy and mitigating discrepancies inherent in full-arch scanning procedures.

Conversely, the control group, employing conventional scan bodies, exhibited mean cRMS values of 244.13 µm, exceeding the established tolerance. This indicates that conventional scan bodies may fail to provide the necessary accuracy for full-arch implant impressions in edentulous scenarios, thereby highlighting their clinical limitations in ensuring precise prosthesis fit and reducing mechanical complications.

In terms of angular deviation, both the experimental and control groups demonstrated mean values of 0.38° and 0.39°, respectively, which are well within the 1° tolerance limit. These results suggest that while AGD implementation does not significantly impact angular deviation on a global scale, localized improvements at specific points (e.g., sites 2.4 and 1.4) may contribute to its overall effectiveness in stabilizing scan body orientation and improving cross-arch accuracy.

Previous studies have consistently highlighted the limitations of conventional scan bodies in full-arch cases, particularly in edentulous scenarios. Research by Mizumoto et al. (24) and Fluegge et al. (25) has shown that long inter-implant distances and the absence of anatomical landmarks lead to stitching errors and cross-arch distortions when using traditional scan bodies. While intraoral scanners have improved workflows, their accuracy remains inadequate in some clinical situations, as reported by Zhang et al. (26). In recent studies, the accuracy of IOSs has been a focal point (27). A comparative analysis of ten IOS devices revealed that while precision remained consistent across scanners, trueness varied, with the Trios series exhibiting superior accuracy. Notably, accuracy diminished over longer scanning distances, and diagonal scanning patterns were less reliable, underscoring the need for meticulous scanning strategies during full-arch procedures. Additionally, factors such as ambient lighting, scanning patterns, and the design and material of the implant scan bodies significantly influence the accuracy of intraoral digital implant scans. Optimizing these parameters, tailored to the specific IOS and clinical scenario, is crucial for enhancing scanning precision (27, 28).

Studies (20, 29–31) have shown that scan bodies made entirely of titanium exhibit higher accuracy compared to those combining PEEK with a titanium base. This suggests that the combination of PEEK with a titanium base may compromise the precision of digital implant impressions. Titanium scan bodies are widely used in digital implant impressions due to their durability and favorable optical properties (20, 30). Their roughened surfaces provide excellent optical characteristics for scanners, enhancing the accuracy of digital impressions. However, studies indicate that the material and diameter of scan bodies can influence scanning precision. For extraoral scanners, regular diameter titanium scan bodies have demonstrated higher precision compared to narrow diameter ones (20, 29–31). Conversely, for intraoral scanners, an inverse relationship was observed, suggesting that the choice of scan body should be tailored to the specific scanning device and clinical scenario.

This study confirms these limitations, as the control group using conventional scan bodies exhibited significant cRMS and TotRMS discrepancies.

In contrast, the novel AGD demonstrated a substantial reduction in cRMS values (*p* < 0.001) and a notable improvement in TotRMS (*p* = 0.002). These results align with the findings of Wu et al. (21), who reported improvements in impression accuracy with the use of auxiliary devices. However, unlike previous studies that employed auxiliary devices requiring additional scanning steps or complex workflows (21, 27, 28), the AGD in this study integrates seamlessly into standard protocols. This highlights its clinical practicality and ease of adoption compared to other auxiliary solutions (32, 33).

Previous research by Revilla-León et al. (34) identified significant angular deviations across all sites when using conventional scan bodies; this study found that the AGD mitigated these deviations at specific points (e.g., points 2.4 and 1.4; *p* = 0.017 and *p* < 0.001, respectively). This suggests that the AGD may provide localized stability improvements, addressing cross-arch inaccuracies that are particularly problematic in full-arch cases.

Canullo et al. (35) reported improved angular deviation with an AGD (0.87° vs. 0.64° without an AGD; *p* = 0.005), while the TotRMS values trended toward better results (52.55 µm with an AGD vs. 50.78 µm without an AGD; *p* = 0.051). These findings highlight AGDs' effectiveness in stabilizing scan bodies and reducing cross-arch inaccuracies.

The meta-analysis by Wan et al. (36) confirmed that AGDs enhance scanning accuracy in edentulous arches by providing artificial landmarks. Five studies (17, 21, 37–39) supported AGD use, showing reduced stitching errors, while three highlighted the importance of strategic AGD placement for optimal trueness. Low-level flat AGD configurations were the most accurate (51.87 ± 6.83 µm), outperforming curved designs and control groups (82.29 ± 17.31 µm; *p* < 0.001) (20, 21).

The enhanced trueness and precision observed with the AGD have critical clinical implications. Accurate impressions are essential for achieving a passive fit, reducing the risk of complications such as screw loosening, prosthesis fractures, and peri-implant stress (40). By minimizing discrepancies in implant positioning, the AGD supports the fabrication of prosthetic frameworks with higher precision (34), streamlining clinical workflows and reducing chairside adjustments during prosthesis delivery (41, 42). This aligns with findings from Patzelt et al., who emphasized the importance of impression accuracy in optimizing digital workflows (43).

Some reflections can be made regarding the scanning strategy. It is important to note that the matching between scan bodies and CAD analogs does not occur along the AGD. For this reason, placing an AGD near contiguous scan bodies may reduce the definition of the scan itself. Further studies are required to determine the optimal scanning strategy and the best positioning of the AGD.

Several limitations should be considered when interpreting the results of this study. First, the investigation was conducted under controlled laboratory conditions, which do not replicate clinical variables such as saliva, patient movement, soft tissue behavior, and humidity, all of which may influence scan accuracy *in vivo*. Second, the study employed only one intraoral scanner (Dexis 3800) and a single type of auxiliary geometric device and scan body system (Nobel Biocare), which limits the generalizability of the findings across different digital systems and manufacturers. Additionally, the absence of operator variability in the scanning procedure may not reflect real-world clinical scenarios, where technique and experience can significantly affect outcomes. Finally, while the model used mimicked an edentulous maxilla, anatomical complexities such as severe ridge resorption or angled implants were not represented. Future clinical studies are needed to validate these findings across diverse patient conditions and digital workflows.

Further investigations should focus on *in vivo* studies to confirm these results under clinical conditions and to evaluate long-term outcomes, such as patient satisfaction and prosthetic durability.

Another avenue for investigation is the integration of AGDs with emerging technologies, such as photogrammetry or AI-based impression systems. Combining these approaches may further enhance accuracy and efficiency, potentially redefining standards in full-arch implant rehabilitation.

## Conclusion

This study highlights the AGD as an effective tool to overcome the limitations of conventional scan bodies in full-arch implant impressions. The AGD significantly improved trueness and precision without adding workflow complexity, enhancing implant positioning and prosthetic fit. These benefits may reduce mechanical complications and improve long-term outcomes. While promising, further *in vivo* research is needed, especially in challenging clinical cases. Future studies should also explore integration with technologies such as photogrammetry and artificial intelligence (AI). Overall, the AGD offers a practical solution to optimize digital workflows and clinical efficiency.

## Data Availability

The raw data supporting the conclusions of this article will be made available by the authors, without undue reservation.
